# Combined Invasive Peripheral Nerve Stimulation in the Management of Chronic Post-Intracranial Disorder Headache: A Case Report

**DOI:** 10.3390/clinpract13010027

**Published:** 2023-02-17

**Authors:** Athanasia Alexoudi, Efstathios Vlachakis, Stamatios Banos, Konstantinos Oikonomou, Panayiotis Patrikelis, Anastasia Verentzioti, Maria Stefanatou, Stylianos Gatzonis, Stefanos Korfias, Damianos Sakas

**Affiliations:** 1 Department of Neurosurgery, National & Kapodistrian University of Athens, Evangelismos Hospital, 10646 Athens, Greece; 2 Neurological Institute of Athens (NIA), Vas. Sofias 51, 10676 Athens, Greece; 3 Royal Victoria Infirmary, Newcastle Upon Tyne Hospitals, NHS Trust, Newcastle NE14LP, UK

**Keywords:** combined peripheral nerve stimulation, occipital nerve stimulation, secondary headache disorders, chronic post-intracranial disorder headache and case report

## Abstract

The introduction of ventricular shunts dramatically changed the outcome and quality of life of hydrocephalic patients. However, shunt surgery continues to be associated with numerous adverse events. Headache is one of the most common complications after shunt operation. It is often of prolonged duration, the symptoms resemble those of migraine, and pain does not respond to medication. We propose invasive peripheral nerve stimulation as a potential solution in the treatment of patients suffering from chronic headache associated with shunted hydrocephalus. A young woman presented with daily holocephalic headache with diffuse pain exacerbated by lying down. Imaging revealed panventricular enlargement and possible aqueduct stenosis. When a ventriculoperitoneal shunt was placed, clinical symptoms resolved. Nevertheless, she gradually exacerbated after a second valve replacement due to wound infection. Imaging revealed decompressed ventricles and appropriate shunt placement. The diagnosis of chronic post-intracranial disorder headache was set. Therefore, occipital nerve stimulation was applied and, considering that the patient did not have a total response, bilateral parietal stimulation was added. Three months after the combined PNS, she experienced total remission of headache. Combined PNS eases refractory headaches much more than occipital nerve stimulation alone and could be considered as a solution for shunted hydrocephalus-associated headache.

## 1. Introduction

The introduction of ventricular shunts dramatically changed the outcome and quality of life of hydrocephalic patients. Despite the continued development of various valve types, shunt surgery continues to be associated with numerous adverse events. Headache, epilepsy, and abdominal pain are the most common complications after shunt operation. They are considered mild to moderate adverse events of which neurosurgeons need to be aware [1]. Headaches in patients with shunts do not necessarily mean shunt failure or malfunction, CSF overdrainage, or even intracranial bleeding. In these cases, complaints of chronic headache are often over a prolonged duration, the symptoms resemble those of migraine, and pain does not respond to medication. Some authors suggested that the concept of shunt migraine should not be overlooked. It was identified that patients with treated idiopathic intracranial hypertension (IIH) had headaches that could be classified by current IHS criteria compatible with episodic and chronic tension-type headache, migraine with and without aura, analgesic overuse headache, idiopathic stabbing headache, and benign exertional headache [2,3,4].

Peripheral nerve stimulation (PNS) of the occipital nerves (ONS) is a procedure that is primary applied to treat pain associated with refractory chronic migraine and chronic cluster headaches. ONS has been shown to affect blood flow in brain structures, interfering with the pathophysiological mechanisms of migraine [5].

To our knowledge, patients with chronic headache associated with shunted hydrocephalus responding to invasive PNS has not been previously reported. We describe the significant response of combined invasive PNS for a 33-year-old woman suffering from chronic post-shunt headache.

## 2. Case Presentation

A 33-year-old Caucasian woman with holocephalic headache presented to the outpatient clinic with a 3-week history of deteriorating headaches. She described diffuse daily pain exacerbated by lying down and relieved by sitting up or standing. She had associated gait impairment and visual symptoms.

Her neurological exam was unrevealing, excluding bilateral papilledema. There was no previous headache history or medication overuse, and no family history of neurologic disorders. The findings of the general medical examination were normal.

**Figure 1:**
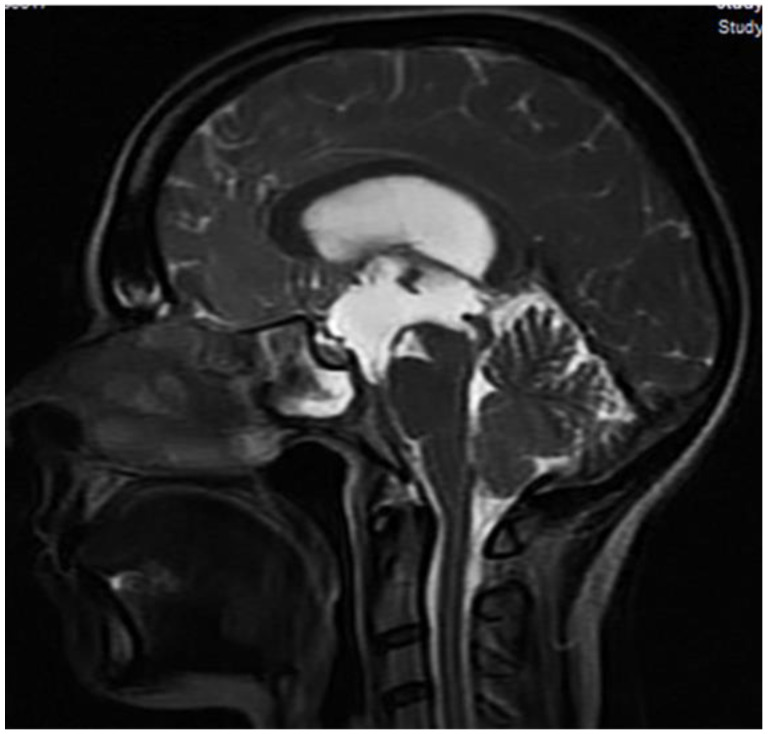
MRI shows Aqueduct stenosis.

Non-enhanced brain magnetic resonance imaging (MRI) revealed ventricular dilatation and possible aqueduct stenosis (Figure 1). 

The patient consented to participate in the intracranial pressure long-term telemetry monitoring protocol, which was held between September 2016 and December 2019. The ethics committee of our hospital approved the study and informed consent was obtained. Thus, she underwent ICP monitoring implantation (via a right frontal ICP bolt insertion—Neurovent^®^ P-tel Raumedic^®^) for a longer period of 2 months. The telemetric ICP monitoring and recording started immediately after implantation in the nursing unit. The ICP recordings were examined during the daytime and nighttime for episodes of raised ICP and the evaluation of ICP wave morphology such as presence of pathologic Lundberg B-waves or A-waves. The trace was analyzed and revealed intracranial hypertension (>30 mmHg). 

A ventriculoperitoneal shunt (VPS) (Medtronic Strata^®^) was placed in the occipital region, which resulted in resolution of her symptoms. A follow-up with a head CT scan also revealed resolution of the hydrocephalus. 

After VPS placement, intracranial pressure values were initially measured close to the upper normal limit (<20 mmHg) with appropriate manipulations in the regulation of the valve mechanism. 

**Figure 2:**
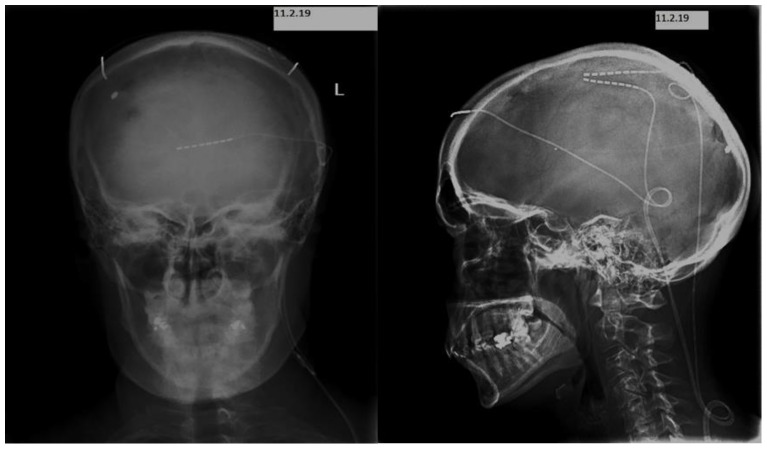
Coronal and sagittal imaging of the placement of three leads with 8 contacts (two leads at the area of parietal lobes symmetrically on both sides and one lead occipital under the superior nuchal line).

One month after the shunt was placed, the patient presented with a cranial wound infection. She was not affected, and there were no changes compared to her previous neurological examination. CSF cultures were negative. The valve cultures grew Staphylococcus aureus sensitive to levofloxacin. She underwent a shunt replacement with a frontal ventricular approach on the contralateral side and she completed a four-week course (Figure 2). 

The patient’s condition gradually deteriorated after the second valve replacement. She complained of a severe daily headache. The headache was a long-lasting, holocephalic, pulsating, exploding headache aggravated by routine physical activity, accompanied by photophobia, phonophobia, and nausea, not responding to common pain medication, influencing her daily activities and the quality of sleep. The underlying organicity was excluded because the headache was not related to pressure and there were no other features and tests suggestive of valve malfunction or overdrainage. Therefore, our patient’s condition met the description of a severe non-aura chronic migraine [6]. The assessment of headache severity and related disability is shown in Table 1. 

A non-contrast head CT scan revealed decompressed ventricles and appropriate VPS placement and intracranial pressure values measured within the normal range (<15 mmHg). 

In our case, a new headache first occurred in close temporal relation to CSF fistula placement and was, therefore, considered either a secondary headache attributable to a nonvascular intracranial disorder or a persistent headache attributable to craniotomy (or, in other words, post-scalp incision chronic headache) [6,7]. A headache attributed to craniotomy may be more diffuse and resemble tension-type headache or migraine [6]. Additionally, this new headache was daily, occurring for more than three months, and had the features of migraine, fulfilling the diagnostic criteria of chronic migraine [6]. Considering the findings of imaging and the intracranial pressure values, other cases of shunt-related headaches (e.g., intracranial hypotension, intermittent proximal obstruction, shunt failure without ventricular enlargement, increased ICP with a working shunt) were excluded [8].

Non-steroid anti-inflammatory drugs (NSAIDs) failed to control the headaches and the response to tramadol was transient. Pharmacological treatment of her headaches with topiramate (100 mg twice daily for 3 months), propranolol (120 mg/daily for 3 months), venlafaxine (150 mg daily for 6 months, in combination with flunarizine), and flunarizine (10 mg daily for 2 months, in combination with flunarizine) proved to be ineffective. The headache did not subside or improve markedly after three classes of migraine prophylactic medications, each used for at least 3 months. The intensity of pain had not been reduced by more than 30% and contributed significantly to a poor quality of life and, as such, was classified as refractory [9]. Most responders to botulinum toxin describe their head being crushed, clamped, or stubbed by external forces, what we understand as imploding headache. We did not consider botulinum toxin injection, because the described exploding headache does not respond to this treatment [10,11]. Anti-CGRP monoclonal antibodies were not in our routine clinical practice when we were handling this case [12].

In patients with chronic medically refractory headaches, including migraine, neuromodulation treatment targeting peripheral nerves is an attractive and valuable approach which offers symptom relief [13]. Considering the clinical characteristics and the refractoriness of symptoms, we considered a trial of peripheral nerve stimulation. The patient provided written informed consent prior to the procedure.

### Implantation Techniques and Devices

The implantation of the peripheral nerve stimulator was performed in two stages. We started with a 2-week trial, and we implanted two electrodes (Boston Scientific Corporation (BSC) Spectra™ System) subcutaneously, bilaterally parietally in the region of the greater occipital nerve territory.

To assess headache severity, disability due to headache, quality of life, insomnia, depression, and anxiety before and after implantation, we used the following rating scales: Visual Analog Scale Score (VAS), Migraine Disability Assessment Scale (MIDAS), Short-Form (36) Health Survey (SF-36), Athens Insomnia Scale (AIS), Beck Depression Inventory (BDI II), and Beck Anxiety Inventory (BAI) [14,15,16,17,18,19]. The MIDAS has not been validated for secondary headaches. However, we decided to include this rating scale in our instruments because the described headache met the diagnostic criteria for chronic migraine. The rating scales were applied 3 months before and 3 months after the final procedure.

The trial was considered successful, as the patient stated at least 50% reduction in pain (the VAS score reduced from 10 to 3). Considering that our patient did not have a total response to occipital nerve stimulation (ONS) and based on the clinical approach of covering the remaining painful frontal area as best as possible, we hypothesized that she would benefit when additional supraorbital stimulation was performed.

**Figure 3:**
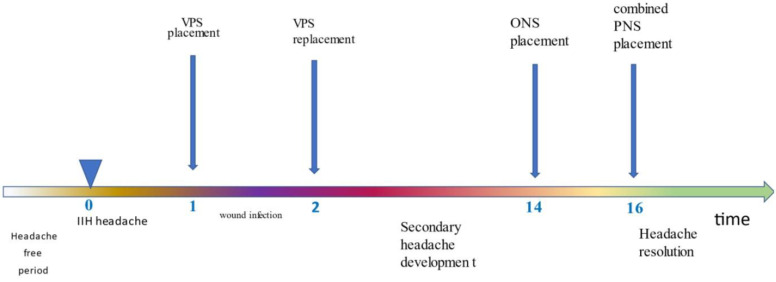
Timeline of events. Time in months.IIH: idiopathic intracranial hypertension, VPS: ventriculoperitoneal shunt, ONS: occipital nerve stimulation, PNS: peripheral nerve stimulation.

For the permanent implant, we decided to apply electrical stimulation by placing leads in the subcutaneous area of the scalp at the point of initiation of daily headache before extending to the whole head. We finally placed three leads with eight contacts: two leads at the parietal lobe areas symmetrically on both sides—more specifically, in the areas of distribution of the greater occipital nerves—and one supraorbital lead (Figure 3).

The device was activated after the surgical site healed, 10 days after implantation. Three months after the implantation of the bilateral combined stimulation system, headache promptly and completely resolved, and quality of life was improved and remained completely medication-free (Table 1). No side effects were observed. When we tried to inactivate the stimulation for a few hours, pain reappeared. The stimulator is continuously on during the day. She uses the single frontal lead and the double parietal system equally. The patient has displayed sustained efficacy with this management over a follow-up period of 4 years 9 (Figure 3).

## 3. Discussion

The patient described here presented with a new-onset daily headache associated with reclination and gait impairment, suggesting increased intracranial pressure. She was diagnosed with hydrocephalus and was successfully treated with VPS. The pathogenesis of hydrocephalus-related headache is correlated with any pathophysiological process capable of causing alterations in CSF production, circulation, and absorption [20].

After the valve revision due to postoperative infection, our patient complained of the development of a new pulsating headache that resembled the characteristics of a non-aura headache. After implantation of the PNS, she experienced total remission of symptoms. These findings led us to speculate that the latter headache may share similar biological mechanisms with a primary headache, such as a migraine. The genesis of a secondary headache attributed to post-intracranial disorder or to craniotomy may also involve peripheral sensitization to neurogenic inflammation as a consequence of sensitization of trigeminovascular afferents. Taylor et al. also proposed this mechanism to explain the genesis of brain tumor headache [21]. These observations provide a new insight into the pathophysiology of secondary headaches, suggesting an overlap with primary headaches.

The convergence of cervical, somatic, and dural afferents on second-order nociceptors in the trigeminocervical complex (TCC) in animal studies provided the theoretical background for the application of ONS [22,23]. This, indeed, explains how the stimulation of the anatomically distant occiput relieves symptoms in patients with certain intractable headaches and a frontotemporal pain distribution. Nevertheless, not all the patients experience adequate relief, and this applies to our case. In these conditions, the stimulation of the supraorbital area produces concordant paresthesia, covering the painful frontal region with stimulator-induced paresthesia. This rationale explains the additional beneficial effect on holocephalic headaches offered by combined PNS [24].

In migraine, neuromodulation approaches regard peripheral nerve stimulation (PNS). They range from non-invasive techniques to surgically implanted devices such as those in occipital nerve stimulation. Invasive PNS has mainly been performed in disabled patients. Current clinical trials suggest the application of invasive (subcutaneous) ONS in occipital neuralgia and in several primary chronic headache types, including chronic cluster headache, chronic migraine, hemicrania continua, and short-lasting unilateral neuralgiform headache attacks with cranial autonomic symptoms (SUNA) [25]. Simultaneous stimulation of the occipital (ONS) and supraorbital nerves (SNS) has been reported to be more successful [24].

However, only a few reports have demonstrated the efficacy of ONS for secondary headache disorders such as cervicogenic headache, C2-mediated headaches, posttraumatic headache, and postsurgical headaches [26,27]. There is a growing field of stimulation devices used in patients with medically intractable primary headache syndromes.

Neurosurgeons are not always familiar with handling chronic, refractory, secondary to shunt placement headaches that are not related to the valve dysfunction. This condition frequently creates exhaustion for the patients themselves, but also for the health system, as health workers are forced to undergo repeated diagnostic tests and inadequate treatments. These different kinds of headaches (e.g., migraines with or without aura, tension-type headache) usually appear several times a month and tend to become chronic and drug-resistant. As known chronic pain conditions are strictly linked with central sensitization, PNS modifies synaptic plasticity, leading to clinically significant and sustained results [28]. Therefore, we have the conviction that PNS could be considered as a possible solution for individuals within this population, who have failed to respond to first-line interventions.

Our patient reported that stimulation rapidly suppressed the pain, but pain recurred when stimulation was inactivated. The main limitation of the current case report is that the results regard only one patient. In addition to the sustained efficacy, the fact that when the stimulation was ceased, the pain reoccurred strengthened the management of this case. Furthermore, our study was not combined with functional or electrophysiological techniques. Therefore, in the absence of a functional or electrophysiological marker, the specific type of modulation that occurred remains unclear.

This report shows a way to deal with these cases and find a path to improve quality of life. The heterogeneity of the pain phenotype is a real challenge in the design of therapeutic studies using neuromodulation techniques and the therapeutic possibilities offered by brain neuromodulation are expanding. Neurostimulation devices should also be used in patients with secondary intractable pain in headache centers to validate the efficacy and safety of the method and be officially suggested as a potential therapy in this patient population. In any case, it is a reversible, adjustable, testable, and safe procedure.

Additionally, we would like to underline the contribution of telemetric ICP recordings, which were useful for the diagnosis confirmation towards appropriate treatment. The direct documentation of the post-adjustment and postoperative ICP normalization was also an advantage.

Our report not only confirms the historical data that suggest combined PNS stimulation facilitates refractory headaches much more than ONS alone, but also suggests common pathophysiological mechanisms between primary and secondary headaches. Furthermore, controlled studies with strict criteria regarding selection of the appropriate candidates for PNS are needed to validate and disseminate the use of neurostimulation in secondary headaches. Finally, once the efficacy of the method is confirmed, a collaboration between neuroscientists and the industry is required towards the optimization of existing devices.

## Figures and Tables

**Table 1:** Pre- and post-implantation performance (3 months before and 3 months after the combined PNS application), regarding the severity of headache, disability due to headache, quality of life, insomnia, depression, and anxiety.

	Pre-Implantation	Post-Implantation
VAS	10	0
MIDAS	90	20
SF36	31	63
AIS	17	6
BDI II	38	24
BAI	26	7

VAS: visual analog scale score, MIDAS: Migraine Disability Assessment Scale, SF36: Short-Form (36) Health Survey, AIS: Athens Insomnia Scale, BDI II: Beck Depression Inventory, BCAI: Beck Anxiety Inventory.

## Data Availability

As the corresponding author, I take full responsibility for the data, analyses and interpretation, and the conduct of the research. I have full access to all data and, subsequently, the right to publish any and all data separately and independently of any sponsor.
